# The chemomodulatory effects of resveratrol and didox on herceptin cytotoxicity in breast cancer cell lines

**DOI:** 10.1038/srep12054

**Published:** 2015-07-09

**Authors:** Ghada A. Abdel-Latif, Ahmed M. Al-Abd, Mariane G. Tadros, Fahad A. Al-Abbasi, Amany E. Khalifa, Ashraf B. Abdel-Naim

**Affiliations:** 1 Department of Pharmacology and Toxicology, Faculty of Pharmacy, Ain Shams University, Egypt; 2 Department of Pharmacology and Toxicology, Faculty of Pharmacy, King Abdulaziz University, Saudi Arabia; 3 Department of Pharmacology, National Research Center, Giza, Egypt; 4 Department of Biochemistry, Faculty of science, King Abdulaziz University, Saudi Arabia; 5 57357 Children's Cancer Hospital, Cairo, Egypt

## Abstract

Herceptin is considered an essential treatment option for double negative breast cancer. Resveratrol and didox are known chemopreventive agents with potential anticancer properties. The aim of the current study is to investigate the influence of resveratrol and didox on the cytotoxicity profile of herceptin in HER-2 receptor positive and HER-2 receptor negative breast cancer cell lines (T47D and MCF-7 cell lines, respectively). The IC_50_’s of herceptin in T47D and MCF-7 were 0.133 ± 0.005 ng/ml and 23.3795 ± 1.99 ng/ml respectively. Equitoxic combination of herceptin with resveratrol or didox in T47D significantly reduced the IC_50_ to 0.052 ± 0.001 and 0.0365 ± 0.001 ng/ml, respectively and similar results were obtained in MCF-7. The gene expression of BCL-xl was markedly decreased in T47D cells following treatment with herceptin/resveratrol compared to herceptin alone. Immunocytochemical staining of HER-2 receptor in T47D cells showed a significant reduction after treatment with herceptin/resveratrol combination compared to herceptin alone. On the contrary, herceptin/didox combination had no significant effect on HER-2 receptor expression. Cell cycle analysis showed an arrest at G2/M phase for both cell lines following all treatments. In conclusion, herceptin/resveratrol and herceptin/didox combinations improved the cytotoxic profile of herceptin in both T47D and MCF-7 breast cancer cell lines.

Breast cancer is a heterogeneous disease that can be classified into different subsets with distinct biology and molecular profiles^1^; some breast cancer phenotypes can be associated with exaggerated tumor aggressiveness and poor clinical outcome^2^. Breast neoplastic disorders vary according to the expression of estrogen receptors , progesterone receptors , and amplification of HER-2 receptors^3^. The phenotypic characteristics of these subgroups are important not only for diagnosis and prognosis, but also as a predictive response to targeted therapies towards these receptors and their underlying signaling pathways.

The HER-2/neu gene encodes a 185-kDA transmembrane tyrosine kinase receptor and belongs to the EGF receptor (EGFR) family^3^.The overexpression of HER-2 has been reported in 20–30% of patients with breast cancer. The overall survival and time to relapse for HER-2 over expressing patients were significantly shorter^4^. In addition, HER-2 overexpression in breast tissues stimulates malignant phenotypic transformation. On the top of that, HER-2 overexpressing tumors are more resistant to standard chemotherapy treatment^5^.

Overexpression of HER-2 enables constitutive activation of growth factor signaling pathways, serving as oncogenic drivers in breast cancer. Two of the main activated pathways are RAS/Raf/MAPK and the phosphatidylinositol 3-kinase (PI3K)–Akt pathways^6^.

Herceptin (trastuzumab) is a recombinant humanized anti-HER-2 monoclonal antibody approved for the treatment of HER-2 overexpressing metastatic breast cancer. Clinical studies have shown that the response rates to herceptin monotherapy in patients with metastatic breast cancer ranged from 12% to 34% for a median duration of 9 months^7^,^8^.

Resveratrol , a naturally occurring phytoalexin, exerts multiple biological effects against a variety of human tumor cell lines^9^. Several studies have revealed that resveratrol is capable of inducing apoptosis and differentiation in many tumor cell lines, such as colon, breast , and T-cell acute lymphoblastic leukemia cancer cells^10^,^11^,^12^. Resveratrol has been shown to exhibit several potential chemoprotective activities in cell and animal models, including inhibition of PI3K/AKT pathway^13^. Our previous work showed the chemomodulatory potential of resveratrol on docetaxel and doxorubicin in solid tumour cells^14^.

Didox is a simple synthetic antioxidant that increases the radiosensitivity of cancer cells by inhibition of the ribonucleotide reductase resulting in a reduction of the Bcl-2 mediated resistance to apoptosis^15^. Didox has shown several chemomodulatory effect to several classic and non-classic anticancer agents such as doxorubicin liver cancer cells, melphalan in multiple myeloma, and cidofovir in nasopharyngeal carcinomas^16^,^17^,^18^ . In our previous study, didox enhanced the anticancer properties of doxorubicin against liver cancer cells and protected from its dose limiting cardiotoxicity^16^.

The combination of herceptin with chemotherapy has greatly improved response rates and increased survival of HER-2 +ve breast cancer patients^19^. However, the combination of herceptin with these conventional chemotherapies is frequently associated with intolerable side effects particularly cardiac adverse effects^20^. These drawbacks stimulated us to investigate the use of new, less toxic agents with potential chemoprotective effects such as, resveratrol and didox as potential adjuvant therapy combined with herceptin. Since many chemopreventive drugs used today are derived from natural products^21^,^22^, and because many natural products are associated with low toxicity, they are potentially excellent candidates for use as chemopreventive agents^23^. These agents are aimed at increasing the efficacy of herceptin as they are known to share the same downstream molecular mechanism and thus may help in improving the efficacy of herceptin, and preventing or delaying relapse in patients with HER-2 +ve tumors. In the current study, we investigated the potential chemo-modulatory effects of resveratrol and didox to herceptin against HER-2 positive and negative breast cancer cell lines.

## Results

### The influence of resveratrol and didox on the efficacy profile of herceptin

**Figure 1:**
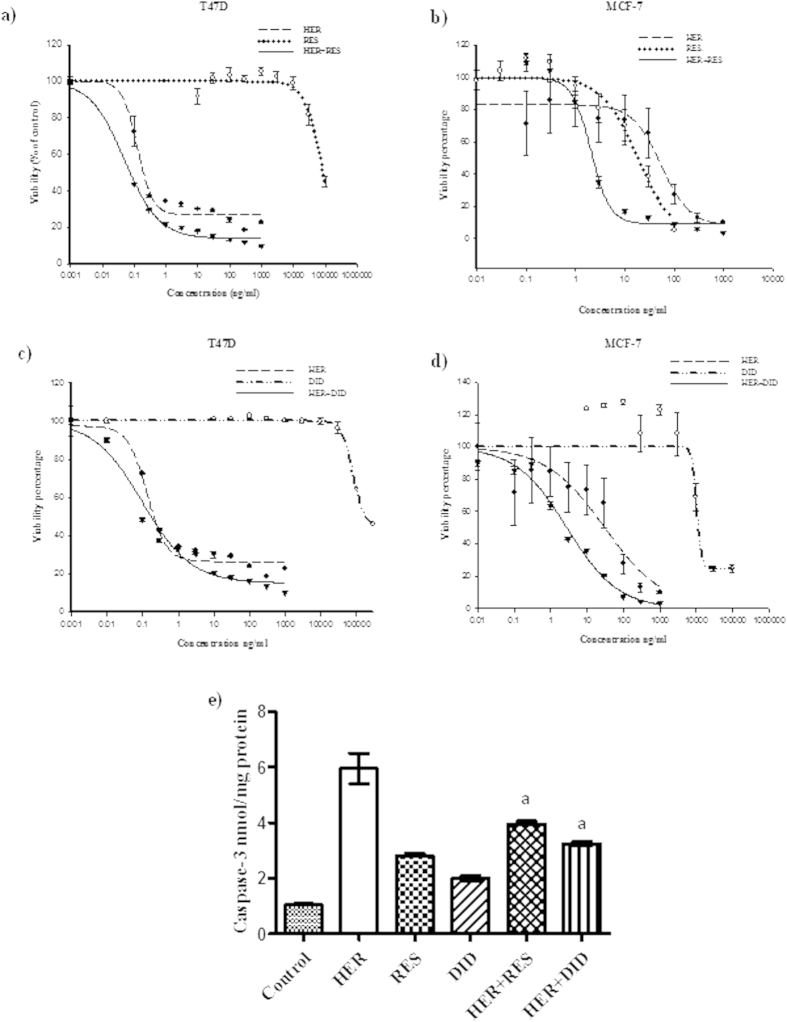
The effect of RES and DID on the dose response curve of HER in T47D (**a**,**c**) and MCF-7 (**b**,**d**) breast cancer cell lines was assessed. Cells were exposed to serial dilution of HER(•) , RES/DID (○) or combination of HER with RES/DID (▼) for 72 h. Cell viability was determined using SRB-U assay. The values on x-axis represent the concentrations of HER, RES (**a**,**b**) or HER, DID (**c**,**d**) single treatments while they represent the concentrations of HER in HER/RES (**a**,**b**) or HER/DID (**c**,**d**) combination treatments. The concentrations of RES and DID in combinations are deduced from the equitoxic ratios of HER/RES (T47D; 1/200, MCF-7; 1/100) and HER/DID (T47D; 1/1000, MCF-7; 1/200).Effect of RES and DID on active caspase-3 level was assessed in HER treated T47D cells (**e**) using ELISA technique. Data are expressed as mean ± S.E. (n = 3). Multiple comparisons were performed using one way analysis of variance (ANOVA) followed by LSD as post hoc test.

To study the effect of resveratrol and didox on the cytotoxic profile of herceptin, the dose response curve of herceptin alone was assessed relative to its combination with resveratrol or didox in two different breast cancer cell lines; MCF-7 and T47D (Table 1). In T47D breast cancer cell line, steeping dose response curve was observed upon treatment with herceptin until resistant fraction of 6.26 ± 0.51% (Fig. 1a,c). IC_50_ of herceptin treatment for 72 h was found to be 0.133 ± 0.005 ng/ml. In the same way, resveratrol and didox single treatments exerted gradual cytotoxicity with increasing concentrations until a resistant fraction of 41.787 ± 2.07% and 43.99 ± 0.21%, respectively (Fig. 1a,c). Both resveratrol and didox produced gradual cellular log kill with IC_50_ of 33.701 ± 2.69 μg/ml with RES and 82.975 ± 5.95 μg/ml with didox. Equitoxic combination of resveratrol significantly improved the IC_50_ of herceptin in T47D cells to about one third of its level in single treatment condition (Table 1). However, resveratrol combination resulted in significant increase in the resistant fraction of T47D cells to 13.66 ± 1.19%. Similarly, equitoxic combination of didox markedly improved the cytotoxic profile of herceptin in T47D cells decreasing IC_50_ of herceptin to about one fifth of its value after single herceptin treatment (Fig. 1a,c). The calculated combination index for herceptin with resveratrol and didox was 0.393 and 0.276, respectively which is indicative of synergistic interaction in T47D cell line (Table 1).

In MCF-7 cells, herceptin exerted gradient cytotoxic activity with increasing concentration until resistant fraction of 6.41 ± 1.3%. Cellular log kill was gradual in profile with IC_50_ of 23.3795 ± 1.99 ng/ml (Fig. 1b,d). Similarly, resveratrol and didox single treatments produced gradual cellular log kill with IC_50_ of 11.994 ± 0.3 μg/ml and 9.506 ± 0.08 μg/ml respectively and a resistant fraction of 9.659 ± 0.14% with resveratrol and 24.187 ± 1.18% with didox (Fig. 1b,d). Equitoxic combination of resveratrol significantly improved the cytotoxic profile of herceptin in MCF-7 cell line; IC_50_ of herceptin after combination with resveratrol was significantly decreased to 1/14 of IC_50_ of herceptin alone treatment (Table 1) but the resistant fraction increased to 8.463 ± 0.16%. Similar to resveratrol, didox improved the cytotoxic profile of herceptin in MCF-7 cell line decreasing the resistant fraction to 5.894 ± 0.04%, IC_50_ of herceptin after combination with didox was significantly decreased to 1/13 of its corresponding value after single herceptin treatment (Fig. 1b,d). The calculated combination index for herceptin with resveratrol and didox was 0.091 and 0.12, respectively which is indicative of strong synergistic interaction characteristics in MCF-7 cell line (Table 1).

### The effect of resveratrol or didox on active Caspase-3 levels in herceptin treated T47D breast cancer cells

To assess the effect of herceptin combination with resveratrol and didox on the proteolytic phase of apoptosis, the levels of active caspase-3 were measured using ELISA technique in T47D cells. The levels of active caspase-3 were significantly increased with all single and combination treatments compared to untreated cells. However, resveratrol and didox could not further increase the activity of caspase compared to herceptin alone treatment (Fig. 1e).

### Effect of resveratrol and didox on herceptin induced apoptosis signaling in breast cell lines

**Figure 2:**
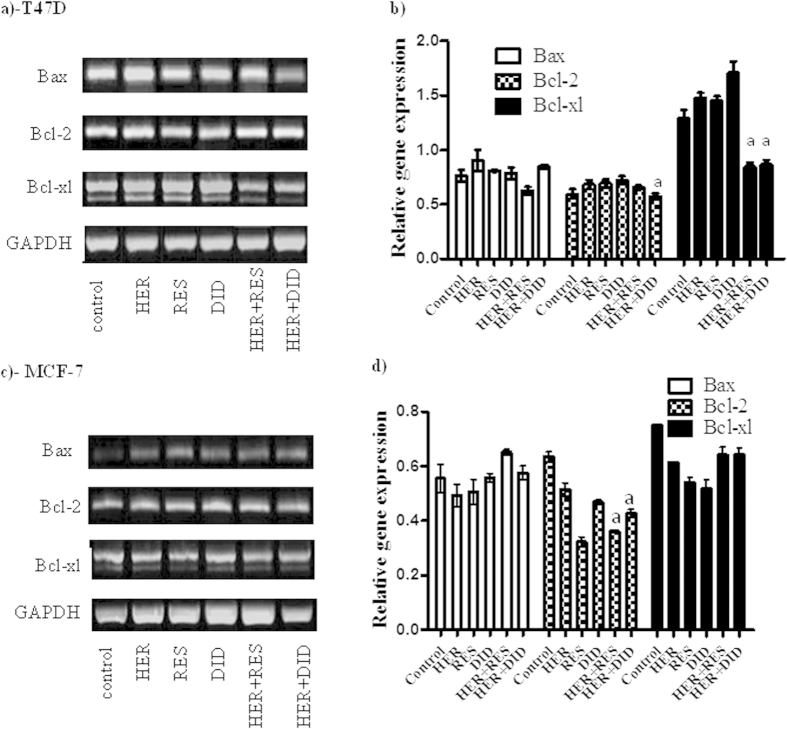
Effect of RES and DID on the apoptosis pathway in HER treated breast cancer cells. Gene expression of Bax, Bcl-2 and Bcl-xl using RT-PCR on T47D (**a** and **b**) and MCF-7 (**c** and **d**) after treatment with HER, RES, DID or their combination was assessed. All samples were derived from the same experiment and gels were processed in parallel. Cropped gels are displayed for comparison between gene expressions in different groups. Full length gels are presented in Supplementary figure 1. Data are expressed as mean ± S.E. (n = 3). Multiple comparisons were performed using one way analysis of variance (ANOVA) followed by LSD as post hoc test.

To explain the interaction characteristics of herceptin with resveratrol and didox, quantitative gene expression analysis for some apoptosis key elements was assessed using real time PCR technique. In T47D cell line, the apoptotic gene, Bax expression was not significantly changed in single treatments compared to the untreated cells. Similarly, no marked change in the Bax expression was observed in all combination treatments compared to single treatment with herceptin (Fig. 2a,b). Reciprocally, Bcl-2 anti apoptotic gene was not significantly over expressed in single herceptin treatment compared to the untreated cells, while a significant decrease in the Bcl-2 expression was observed following herceptin/didox combination compared to single herceptin treatment (Fig. 2a,b). However, Bcl-2 expression was not markedly decreased following herceptin/resveratrol combination compared to herceptin single treatment (Fig. 2a,b). The anti apoptotic gene, Bcl-xl was not significantly overexpressed with all single treatments, however, a marked decrease in the expression of Bcl-xl was observed after combinations, compared to single herceptin treatment (Fig. 2a,b). In MCF-7 cell line, Bax was markedly over expressed in all single treatments compared to untreated cells, and combination treatments compared to single treatment with herceptin (Fig. 2c,d). Bcl-2 expression was significantly decreased in all single treatments compared to untreated cells, and combination treatments compared to single treatment with herceptin (Fig. 2c,d). On the other hand, Bcl-xl expression was not significantly changed in the combination treatments compared to the single treatment with herceptin. However, Bcl-xl expression was apparently decreased in all single treatments compared to untreated cells (Fig. 2c,d).

### The effect of resveratrol or didox on the expression of HER-2 receptor in T47D breast cancer cells

**Figure 3:**
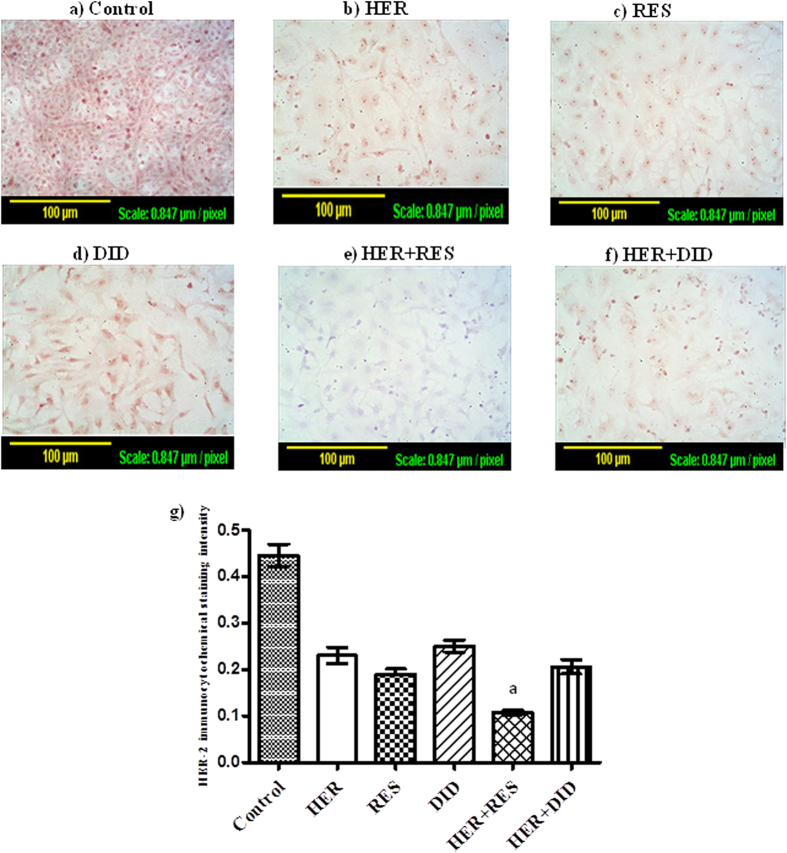
Effect of RES and DID on the HER-2 receptor expression in HER treated T47D breast cancer cells. Immunocytochemical staining of HER-2 recptor in T47D was performed and the optical density was measured after treatment with HER (**b**), RES (**c**), DID (**d**), HER + RES (**e**), HER + DID (**f**) and compared to control cells (**a**). Data are expressed as mean ± S.E. (n = 3). Multiple comparisons were performed using one way analysis of variance (ANOVA) followed by LSD as post hoc test.

To explain the interaction characteristics of herceptin with resveratrol or didox, the expression of HER-2 receptor was determined by measuring the optical density after immunocytochemical staining of the receptor. The expression of HER-2 receptor was significantly decreased after all single and combination treatments compared to the untreated cells (Fig. 3). Combination of herceptin with resveratrol showed a marked reduction of the HER-2 receptor expression compared to single treatments (Fig. 3b,e & g) while combination of herceptin with didox produced no apparent change in the expression of HER-2 receptor (Fig. 3b,f & g).

### The effect of resveratrol and didox combination with herceptin on the cell cycle distribution breast cancer cells

DNA flow-cytometry was used to assess the effect of herceptin, resveratrol, didox and their combination on the cell cycle distribution of T47D and MCF-7 breast cancer cell lines.

**Figure 4:**
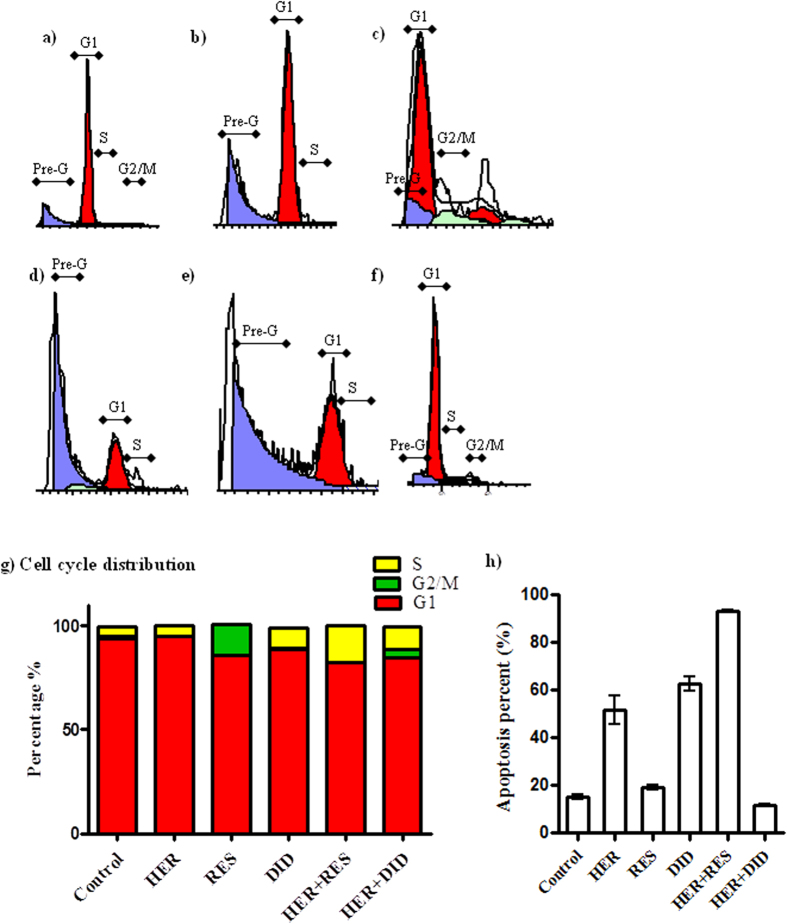
Effect of RES and DID on the cell cycle distribution profile in HER treated T47D breast cancer cells. The cells were exposed to HER (**b**), RES (**c**), DID (d), HER + RES (**e**), and HER + DID (f) for 72 hrs and compared to control cells (**a**). Cell cycle distribution was determined using DNA cytometry analysis, the histograms a,b,c,d,e&f represent the DNA content of the cells in each of the phases of the cell cycle (G1, S & G2/M ) as well as the apoptotic cells (pre-G). Different cell phases were plotted (**g**) as percentage of total events. The apoptotic cells were plot separately (**h**) as percentage of the total events. (n = 3).

In T47D cell line, herceptin produced no significant change in the non-proliferating cell fraction (Go/G1 phase) compared to the control cells (Fig. 4a,b & g), while both resveratrol and didox significantly decreased the non-proliferating cell fraction (Go/G1 phase) from 94.026 ± 1.1% to 85.7 ± 1.2%, and 88.83 ± 1.8% respectively (Fig. 4a,c,d & g). Combination of herceptin with resveratrol and didox apparently decreased the non-proliferating cell fraction from 94.7 ± 0.1% to 82.31 ± 0.4% and 84.64 ± 0.8% respectively, compared to herceptin single treatment (Fig. 4b,e,f & g). No apparent change in the S-phase cell population was observed with herceptin single treatment (Fig. 4a,b & g) while, resveratrol markedly decreased the S-phase cell population from 4.9 ± 0.4% to 0% compared to the control cells (Fig. 4a,c & g). On the other hand, treatment with didox caused a significant increase in the S-phase cell population (10 ± 0.9%) compared to control cells (Fig. 4a,d & g). Reciprocally, a significant compensatory increase in the S-phase cell population was observed after combination of herceptin with resveratrol and didox (17.7 ± 0.4% & 10.86 ± 0.7%) respectively, compared to herceptin alone (Fig. 4b,e,f & g). No significant change in the G2/M phase was observed after treatment with herceptin or didox, compared to control cells (Fig. 4a,b,d & g). On the contrary, treatment with resveratrol caused a significant increase in the G2/M phase to 15 ± 1% compared to control cells (0.8 ± 0.01%) (Fig. 4a,c & g). No apparent change in the G2/M phase was observed after combination of herceptin and resveratrol (Fig. 4b,e & g) while a significant increase in the G2/M phase was observed after combination of herceptin and didox (4.2 ± 0.2%) compared to herceptin alone (0%) (Fig. 4b,f & g). Treatment with herceptin or didox apparently increased the apoptotic Pre-G phase cell population from 15.2 ± 0.7% to 51.4 ± 3.5% & 62.5 ± 1.7% respectively, while treatment with resveratrol caused no significant change in the apoptotic Pre-G phase cell population compared to control cells (Fig. 4a,b,c,d,g & h). A marked increase in the apoptotic Pre-G phase cell population to 93.2 ± 0.3% was observed after the combination of herceptin and resveratrol while combination of herceptin and didox caused a significant decrease in Pre-G phase cell population to 11.63 ± 0.2% compared to herceptin alone (Fig. 4b,e,f, g & h).

**Figure 5:**
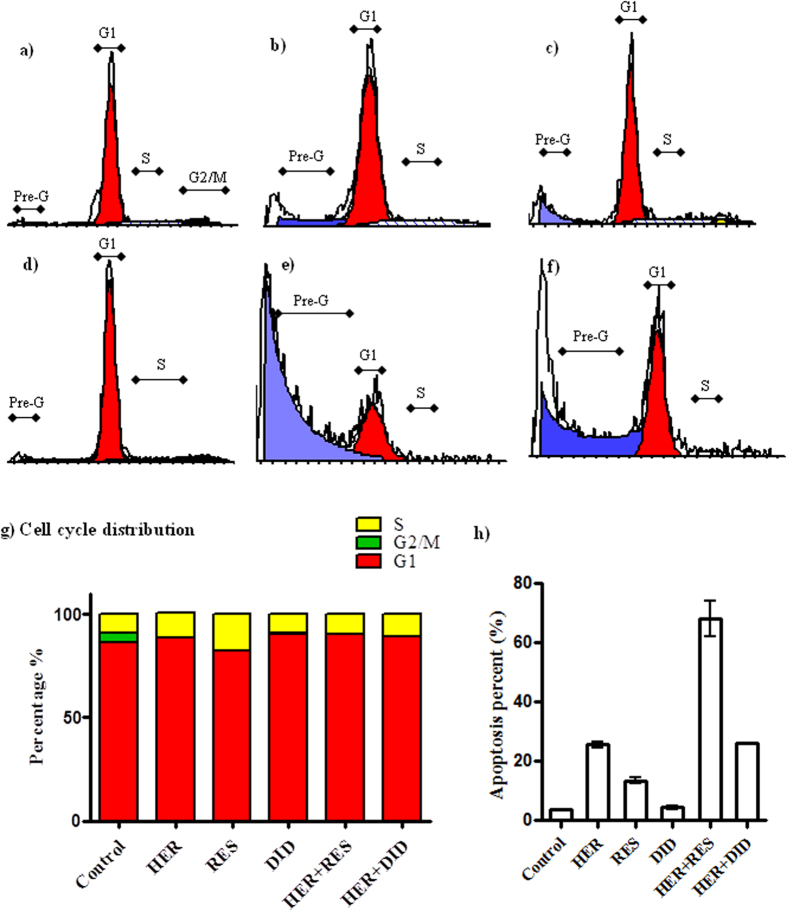
Effect of RES and DID on the cell cycle distribution profile in HER treated MCF-7 breast cancer cells. The cells were exposed to HER (**b**), RES (**c**), DID (**d**), HER + RES (**e**), and HER + DID (**f**) for 72 hrs and compared to control cells (**a**). Cell cycle distribution was determined using DNA cytometry analysis, the histograms a,b,c,d,e&f represent the DNA content of the cells in each of the phases of the cell cycle (G1, S & G2/M ) as well as the apoptotic cells (pre-G). Different cell phases were plotted (**g**) as percentage of total events. The apoptotic cells were plot sepretately (**h**) as percentage of the total events. (n = 3).

In MCF-7 cell line, herceptin produced no apparent change in the non-proliferating cell fraction (Go/G1) (Fig. 5a,b & g) while, resveratrol caused a moderate decrease in the non-proliferating cell fraction (Go/G1) from 86.4 ± 0.3% to 82.4.2 ± 0.2% (Fig. 5a,c & g). On the other hand, a significant increase in the non-proliferating cell fraction was produced by didox (90.6 ± 0.2%) compared to control cells (Fig. 5a,d & g). Combination of herceptin with resveratrol and didox produced no significant change in the non-proliferating cell fraction (Go/G1) phase compared to herceptin alone (Fig. 5b,e,f & g). Treatment with herceptin or resveratrol caused a significant increase in the S phase cell population (11.8 ± 0.8% & 17.6 ± 0.2%) respectively, on the other hand, treatment with didox caused no marked change in the S phase cell population compared to control cells (9.3 ± 0.5%) (Fig. 5a,b,c,d & g). Combination of herceptin and resveratrol caused an apparent decrease in the S phase cell population (9.5 ± 0.2%) (Fig. 5b,e & g), while combination of herceptin and didox caused no significant change in the S phase cell population (10.6 ± 0.2%) compared to herceptin alone (Fig. 5b,f & g). Herceptin , resveratrol and didox markedly decreased the G2/M phase from 4.3 ± 0.2% to 0% with herceptin and resveratrol and 0.3 ± 0.01% with didox compared to control cells (Fig. 5a,b,c,d & g). Combination of herceptin and resveratrol or didox caused similar apparent decrease in the G2/M phase to 0% as herceptin alone (Fig. 5b,e,f & g). A marked increase in the apoptotic Pre-G phase cell population was observed following treatment with herceptin or resveratrol from 3.5 ± 0.1% to 25.5 ± 0.5% and 13.28 ± 0.6% respectively, while didox caused no significant change in the apoptotic Pre-G phase cell population (4.34 ± 0.4%) compared to control cells (Fig. 5a,b,c,d, g & h). Combination of herceptin with resveratrol caused a significant increase in the apoptotic Pre-G phase cell population (68.05 ± 3.5%) (Fig. 5b,e,g & h). While combination of herceptin with didox caused no significant change in the Pre-G phase cell population (26.03 ± 0.1%) compared to herceptin alone. (Fig. 5b,f,g & h).

## Discussion

Overexpression of the HER-2 receptor, occurs in approximately 25% of breast cancer patients and is associated with shorter survival^24^. Trastuzumab (Herceptin®) has become a standard of care for the treatment of HER-2 overexpressing early stage and metastatic breast cancers. However, clinical response rate of HER-2 +ve patients to single herceptin treatment is only 15–30% response rate^25^,^26^, which can be significantly increased to 50–80% by the addition of another chemotherapeutic agent^27^,^28^. In the current study, two potential chemomodulatory drugs (resveratrol and didox) were chosen to improve the anticancer profile of herceptin.

In the current study the combinations of resveratrol and didox with herceptin were tested in two breast cancer cell lines; T47D (HER-2 +ve) and MCF-7 (HER-2 −ve). Both resveratrol and didox synergized the efficacy of herceptin in both cell lines regardless of HER-2 receptor expression. Interestingly, combination of resveratrol and didox with herceptin resulted in stronger synergism (lower CI values and greater reduction in the IC_50_’s) with MCF-7 cells (HER −ve cells) compared to T47D cells (HER +ve cells). Our previous work showed that the combination of resveratrol with doxorubicin and docetaxel resulted in synergistic effect in MCF-7 cell line^14^. Other investigators showed similar results when combining resveratrol with paclitaxel in MCF-7, non Hodgkin lymphoma and multiple myeloma cell lines^29^,^30^. In addition, additive effect was noticed following the combination of resveratrol with rapamycin in MCF-7 cell line^31^. To the best of our knowledge, this is the first time to test the combinations of herceptin with resveratrol and didox in HER −ve and HER +ve breast cancer cell lines. Accordingly, the greater synergistic interaction of resveratrol and didox with herceptin in MCF-7 cells (HER −ve) compared to T47D cell (HER +ve)might be attributed to the activity of the adjuvant agents rather than herceptin.

The effects of herceptin/resveratrol and herceptin/didox combinations on some of the apoptosis key markers (Bax, Bcl-2 and Bcl-xl) were further evaluated. Both combinations of herceptin/resveratrol and herceptin/didox in T47D only decreased the expression of Bcl-xl anti-apoptotic gene. On the other hand, both herceptin/resveratrol and herceptin/didox combinations in MCF-7 increased Bax gene expression and decreased Bcl-2 gene expression while Bcl-xl remains unaffected. Yet, the higher synergistic effects of herceptin/resveratrol and herceptin/didox combinations in MCF-7 compared to T47D might be partly explained by affecting more elements in Bax/Bcl-2/Bcl-xl apoptosis pathway. HER-2 blockade by herceptin is known to attenuate Bcl-2/Bcl-xl expression and push breast cancer cells towards apoptosis^32^. In addition, antiproliferative effect of resveratrol and didox in breast, prostate, and colon cancers is reported to be via suppressing Bcl-2 and Bcl-xl genes^33^. Resveratrol and its analogue HS-1793 induced apoptosis in MCF-7 and MDA-MB-231 breast cancer cell lines by interfering with Bcl-2 gene expression^34^. In our previous work, resveratrol enhanced the cytotoxic profile of docetaxel and doxorubicin in different tumors cells via suppressing Bcl-2 and Bcl-xl gene expressions^14^. This fortifies our assumption that the synergism in MCF-7 cell line between herceptin and either of resveratrol or didox in MCF-7 is attributed to the adjuvant drug.

HER-2 receptor expression is very important for the survival and proliferation of HER +ve breast cancer cells, herceptin blocks HER receptor and suppress its downstream signaling ultimately leading to apoptosis and cytotoxicity^35^. In the current work, single treatment of T47D breast cancer cells with herceptin, resveratrol or didox caused a significant reduction in the expression of HER-2 receptor. Herceptin treatment is repeatedly reported to be associated with the down-regulation of HER-2 receptors^36^,^37^,^38^ . According to our current observation as well as other investigators, treatment with resveratrol was associated with down-regulation of HER-2/neu gene expression in several tumor cells types^39^. Herein, herceptin/resveratrol combination showed superior HER-2 receptor down-regulation compared to single herceptin treatment, while herceptin/didox combination didn’t further depress HER-2 receptor expression compared to herceptin alone. Accordingly, the modest synergism between herceptin and resveratrol in T47D cells might be explained by sharing both agents the same target pathway to induce apoptosis sparing no more room for a stronger combined effect compared to each agent alone. On the other hand, the stronger synergism for herceptin/resveratrol and herceptin/didox combinations in MCF-7 might be attributed to inducing apoptosis via two different pathways; one of them would be Bax/Bcl-2/Bcl-xl axis. The other pathway is probably due to the effect of herceptin on the cancer stem cells subpopulation. The concept of cancer stem cells was introduced by Max S. Wicha and co-workers^40^. Yet, Ithimakin *et al*., identified a subpopulation of cancer stem cells within MCF-7 cell line which uniquely express HER-2 receptors. This stem cell subclone is responsible for the self renewal of the cancer cells^41^.

Herein, the combination of herceptin/resveratrol and herceptin/didox could not further increase the activity of caspase-3 in T47D cells compared to herceptin alone. It is reported previously that herceptin alone and in combination increase intracellular caspase-3 level^42^. On the other hand, apoptosis in MCF-7 cell line is reported to be caspase-3 independe as itlacks caspase-3 expression^43^. Resveratrol and didox induce apoptosis in different tumor cell lines via caspase-dependent and independent pathways^18^,^44^.

In the same line with our previous results, significant increase of cells in late apoptotic phase (pre-G phase) after treatment with herceptin, didox and herceptin/resveratrol was observed in T47D cell. While MCF-7 cells, treatment with herceptin, resveratrol and herceptin/resveratrol combination showed significant increase in the pre-G fraction. This might be secondary to the cell cycle arrest in the S-phase by resveratrol and the arrest in G_0_/G_1_ phase by didox. Singh *et al*., showed that cell cycle arrest in S-phase is accompanied by an increase in the pre-G fraction of MCF-7 cells after treatment with resveratrol^45^. Our previous work didox enhanced doxorubicin cytotoxicity in liver cells due to the induction of cell cycle arrest in S-phase^16^.

In conclusion, both resveratrol and didox synergistically interact with herceptin in T47D and MCF-7 breast cancer cell lines and this synergism is not restricted to HER-2 overexpressing breast cancer cells.

## Materials and Methods

### Drugs and chemicals

Didox was generously gifted from Professor Howard L. Elford, Molecules for Health Inc., Richmond, VA, USA. Herceptin, resveratrol, and sulforhodamine were purchased from Sigma Chemical Co. (St. Louis, MO). RPMI-1640, fetal bovine serum and other cell culture materials were purchased from Lonza Group Ltd. (Basel, Switzerland). Other reagents were of the highest analytical grade.

### Cell culture

Two human breast cancer cell lines were used; T47D (HER-2 +ve) and MCF-7 (HER-2 −ve). Cell lines were obtained from the Vaccera (Giza, Egypt) and maintained in RPMI-1640 containing 100 U/mL penicillin, 100 ug/mL streptomycin , 0.025 ug/mL amphotericin B, supplemented with 10% heat-inactivated fetal bovine serum (FBS). Cell lines were incubated under standard conditions in humidified 5% (v/v) CO_2_ atmosphere at 37 °C.

### Cytotoxicity assay

The cytotoxicity of herceptin, resveratrol and didox were tested against T47D and MCF-7 using the sulforhodamine B (SRB) colorimetric assay for cytotoxicity screening. Exponentially growing cells were collected using 0.25% Trypsin-EDTA and seeded in 96-well plates at 1000–2000 cells/ well. Cells were treated with herceptin, resveratrol and didox and incubated for 72 hours, then fixed with TCA (10%) for 1 hr at 4 °C. After several washings with water 0.4% SRB was added, kept for 10 minutes in the dark and subsequently washed with 1% glacial acetic acid. The plates were left to dry overnight, then Tris-HCl was used to dissolve the SRB stained cells and the colour intensity was measured at 540 nm^46^.

### Data analysis

The dose-response curves were analyzed using the E_max_ model (Eq. 1)^47^.
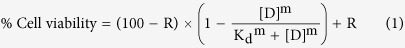
Where R is the residual unaffected fraction (resistance fraction); [D] is the drug concentration used, K_d_ is the drug concentration that produces 50% reduction of the maximum inhibition rate, and m is a Hill-type coefficient. IC_50_ was defined as the drug concentration that produces 50% reduction in the color intensity compared to that of the control (i.e., K_d_ = IC_50_ when R = 0 and E_max_ = 100−R). The IC_50_’s and the R fraction values of herceptin, resveratrol, didox and their combinations were back calculated by substitution in the equation of the regression lines representing the dose-response curves (Eq. 1).

Combination index (CI) was calculated as previously described. The exponentially growing cells were exposed to equitoxic concentrations of herceptin and resveratrol or herceptin and didox in 96-well plates for 72 hrs, and subsequently subjected to SRB assay. Equitoxic concentrations of herceptin and resveratrol or didox were calculated according to the ratio between their IC_50_’s.

CI was calculated from the formula:
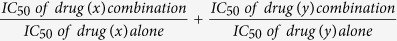
The nature of drug interaction is defined as synergism if CI < 0.8; antagonism if CI > 1.2; and additive if CI ranges from 0.8–1.2^48^.

### RNA extraction, Real time PCR analysis and quantification of gene expression

To assess the gene expression of bax, bcl-2 and bcl-xl following treatment of cells with herceptin, resveratrol, didox and their combination, total RNA extraction from cells was performed using RNeasy Mini Kit® (Qiagen Inc. Valencia, CA, USA). Reverse transcription was undertaken to construct cDNA library from different treatments using High-Capacity cDNA Reverse Transcription Kit (Applied Biosystems, Foster City, CA). The archived cDNA libraries were then subjected to quantitative real time PCR reactions^49^ using cyber green fluorophore (Fermentas Inc., Glen Burnie, MD, USA). Primer sequences were as follows: Bcl-2 forward primer GGG-TAC-GAT-AAC-CGG-GAG-AT and reverse primer CTG-AAG-AGC-TCC-TCC-ACC-AC; Bax forward primer TCT-GAC-GGC-AAC-TTC-AAC-TG and reverse primer TGG-GTG-TCC-CAA-AGT-AGG-AG; Bcl-xl forward primer GGC GGA TTT GAA TCT CTT TCT C and reverse primer TTA TAA TAG GGA TGG GCT CAA CC; GAPDH was used as reference housekeeping gene with forward primer TGC-ACC-ACC-AAC-TGC-TTA-G and reverse primer GAT-GCA-GGG-ATG-ATG-TTC^49^.

### Determination of caspase-3 activity

To determine the effect of herceptin, resveratrol, didox and their combination on apoptosis, the active caspase-3 level was measured by using Quantikine -Human active Caspase-3 Immunoassay (R&D Systems, Inc. Minneapolis, USA) according to the manufacturer protocol. Briefly, cells were washed with PBS,collected and added to the extraction buffer containing protease inhibitors (1 mL per 1 × 10^7^ cells.) then diluted immediately prior to the assay. After performing all steps of the assay the optical density of each well was determined within 30 minutes using a microplate reader set at 450 nm.

### Immunocytochemical determination of HER-2 receptor

The effect of herceptin, resveratrol, didox and their combination on the her-2 receptor expression was determined by immunocytochemical (ICC) staining of her-2 receptor. The cells were grown on glass coverslips that were previously coated with poly L-lysin and then dried and sterilized in the UV. Then the cells were fixed in 50% methanol at 4 °C for 1 hr. Cells were then washed and immersed in TBS (tris buffer saline) to adjust the pH, this is repeated between each step of the ICC procedure. Permealization was done by immersing slides in 3% hydrogen peroxide for 10 min. Power Stain^TM^ 1.0 Poly HRP DAB Kit (Genemed Biotechnologies, CA-USA) was used to visualize any antigen-antibody reaction in the cells. Two drops of the ready to use mouse monoclonal c-erbB-2/HER-2/neu Ab-17 (clone e2-4001+3B5) (Thermo Scientific-cat#MS-730-PCS) were applied to each slide. Subsequently, slides were incubated in the humidity chamber overnight. Henceforward, poly HRP enzyme conjugate was applied to each slide for 30 minutes. DAB chromogen was prepared and 2–3 drops were applied on each slide for 2 min. DAB was rinsed, after which counterstaining with Mayer Hematoxylin and cover slipping were performed as the final steps before slides were examined under the light microscope.

### Analysis of cell cycle distribution

To determine the effect of herceptin, resveratrol, didox and their combinations on the cell cycle distribution effect in MCF-7 and T47D cell lines; cell cycle analysis was performed using the CycleTEST™ PLUS DNA Reagent Kit (Becton Dickinson Immunocytometry Systems, San Jose, California, USA). Control cells with known DNA content (PBMCs) were used as a reference point for determining the DI (DNA Index) for the test samples. The cells were stained with propodium iodide stain following the procedure provided by the kit and then run on the DNA cytometer. Cell cycle distribution was calculated using CELLQUEST software (Becton Dickinson Immunocytometry Systems, San Jose, California, USA).

### Statistical analysis

Data are presented as mean ± SE. Analysis of variance (ANOVA) with LSD post hoc test was used for testing the significance using SPSS^®^ for windows, version 17.0.0. The level p < 0.05 was taken as the criterion for significance.

## Additional Information

**How to cite this article**: Abdel-Latif, G. A. *et al*. The chemomodulatory effects of resveratrol and didox on herceptin cytotoxicity in breast cancer cell lines. *Sci. Rep*. **5**, 12054; doi: 10.1038/srep12054 (2015).

## Supplementary Material

Supplementary Information

## Figures and Tables

**Table 1:** Effect of RES and DID on the cytotoxicity parameters of HER in breast cancer cell lines.

	T47D			MCF-7		
	IC_50_*	R-fraction (%)	CI-value	IC_50_*	R-fraction (%)	CI-value
**HER**	0.13 ± 0.005	6.264 ± 0.51	–	23.38 ± 1.99	7.75 ± 0.1	–
**HER** **+** **RES**	0.05^a^ ± 0.001	13.66^a^ ± 1.19	0.39	1.78^a^ ± 0.08	8.46^a^ ± 0.16	0.09
**HER** **+** **DID**	0.04^a^ ± 0.001	16.94^a^ ± 0.74	0.27	1.82^a^ ± 0.05	5.89^a^ ± 0.04	0.12

^*^The IC_50_ is calculated in ng/ml. The values of the IC_50_ represent the IC_50_’s of HER in single and combination treatments. The values of the IC_50_’s of RES are deduced from the equitoxic ratios of HER/RES in T47D (1/200) and in MCF-7 (1/100).

^a^Significantly different from HER single treatment at P < 0.05.

The values of the IC_50_’s of DID are deduced from the equitoxic ratios of HER/DID in T47D (1/1000) and in MCF-7 (1/200). Multiple comparisons were achieved using one way analysis of variance (ANOVA) followed by LSD post hoc test.
