# Supplementary material for: The chemomodulatory effects of resveratrol and didox on herceptin cytotoxicity in breast cancer cell lines
Source: Sci Rep. 2015 Jul 9;5:12054. doi: 10.1038/srep12054 (PMC4496837; doi:10.1038/srep12054)
Supplement: Supplementary Information [file srep12054-s1.pdf]

The chemomodulatory effects of resveratrol and didox on herceptin cytotoxicity in breast cancer cell lines.

Ghada A. Abdel-Latif[1], Ahmad M. Al-Abd[2, 3*], Mariane G. Tadros[1], Fahad A. Al-Abbasi[4], Amany E. Khalifa[1,5], Ashraf B. Abdel-Naim[1].

[1] Department of Pharmacology and Toxicology, Faculty of Pharmacy, Ain Shams University, Egypt

[2] Department of Pharmacology and Toxicology, Faculty of Pharmacy, King Abdulaziz University, Saudi Arabia

[3] Department of Pharmacology, National Research Center, Giza, Egypt

[4] Department of Biochemistry, Faculty of science, King Abdulaziz University, Saudi Arabia.

[5] seconded as strategic planning consultant at 57357 children cancer hospital, Cairo, Egypt.

*Correspondence to:**

Ahmed M. Al-Abd, Pharmacology and Toxicology Dept. Faculty of Pharmacy, King Abdulaziz University, Jeddah 21589, P.O. 80260

Tel: +966-(0)2-640-0000/Ext 21125

Fax: +966-(0)2-695-1696

E-mail: amalabd@kau.edu.sa

T47D

GAPDH BAX BCL-2 BCL-xl

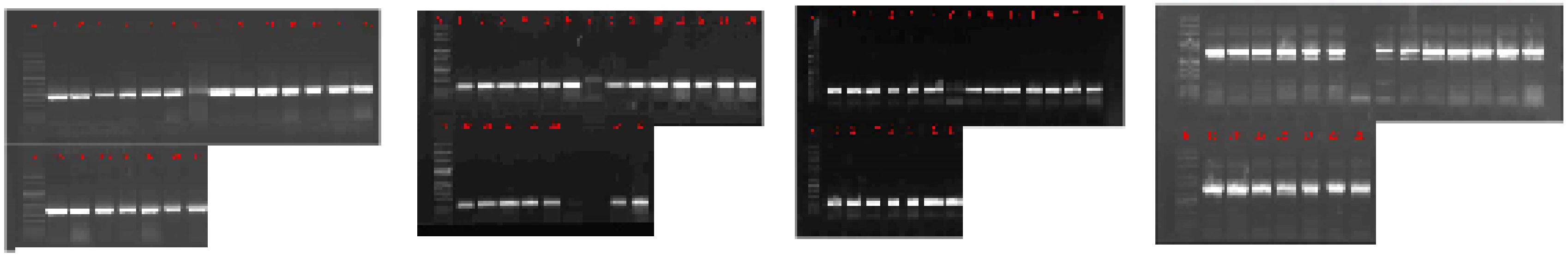

Key for T47D :

From 1-4: control ,From 5-8: DID ,From 9-11: HER ,From 12-15: RES ,From 16-18: HER+DID ,From 19-21: HER+RES

MCF-7

GAPDH BAX BCL-2 BCL-xl

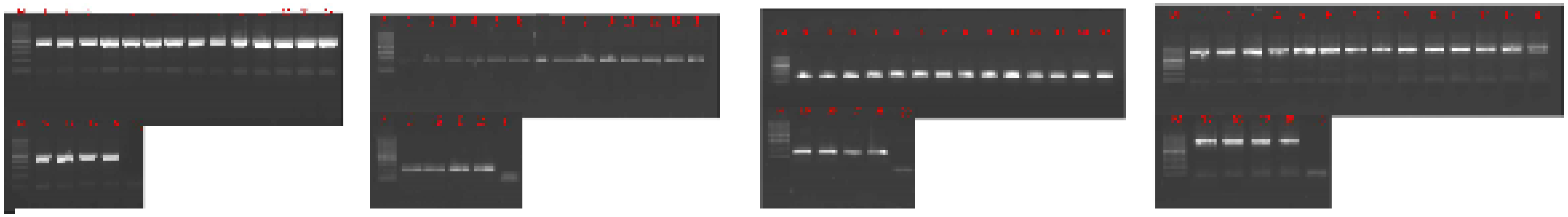

Key for MCF-7:

From 1-3: control ,From 4-6: HER ,From 7-9: DID ,From 10-12: RES ,From 13-15: HER+DID ,From 16-18: HER+RES,(-) NTC
